# Metabolic Reprogramming Triggered by Fluoride in U-87 Glioblastoma Cells: Implications for Tumor Progression?

**DOI:** 10.3390/cells14110800

**Published:** 2025-05-29

**Authors:** Wojciech Żwierełło, Agnieszka Maruszewska, Marta Skórka-Majewicz, Agata Wszołek, Izabela Gutowska

**Affiliations:** 1 Department of Medical Chemistry, Pomeranian Medical University in Szczecin, 70-111 Szczecin, Poland; marta.skorka.majewicz@pum.edu.pl; 2 Institute of Biology, University of Szczecin, 71-415 Szczecin, Poland; agnieszka.maruszewska@usz.edu.pl (A.M.); agata.wszolek@usz.edu.pl (A.W.)

**Keywords:** fluorides, gliomas, oxidative stress, inflammation, tumor microenvironment

## Abstract

Chronic inflammation is a hallmark of brain tumors, especially gliomas, which exhibit elevated levels of pro-inflammatory mediators within the tumor and its microenvironment. Metabolic disturbances triggered by fluoride as a pro-oxidative agent in glioma cells, known for their high aggressiveness and resistance to therapy—remain poorly understood. Therefore, investigating the impact of physiologically elevated fluoride concentrations on oxidative stress and pro-inflammatory responses in glioma cells represents a relevant and timely research objective. Methods: U-87 human glioblastoma cells were subjected to short-term and long-term exposure to physiologically high concentrations of NaF (0.1–10 µM). Both the cells and the culture medium were analyzed. We assessed levels of reactive oxygen species (ROS), antioxidant defenses, and a panel of cytokines and chemokines. Results: Our results demonstrated that oxidative stress and inflammatory conditions in U-87 cells varied with fluoride concentration and exposure time. This led to an increase in ROS levels and key pro-inflammatory cytokines, including IL-6 and TNF-α. Conclusions: Fluoride compounds can generate ROS and disrupt the antioxidant defense system in U-87 human glioblastoma cells, leading to the initiation and progression of inflammatory states. Furthermore, prolonged exposure to NaF may induce adaptive mechanisms in U-87 cells.

## 1. Introduction

Inflammatory signaling in glioblastoma (GBM) cells plays a crucial role in the pathogenesis and progression of this aggressive brain tumor. The inflammatory process in GBM is driven by complex interactions between tumor cells and the tumor microenvironment, which involve changes in the concentration and activity of numerous cytokines and chemokines, including IL-6, IL-1β, and tumor necrosis factor α (TNF-α). These molecules serve as key regulators of inflammation in GBM [1,2]. This process is often accompanied by microglial activation, which supports the secretion of pro-inflammatory cytokines and the generation of reactive oxygen species (ROS), further stimulating tumor invasiveness [3]. In gliomas, microglia frequently adopt the M2 phenotype, which promotes angiogenesis and tumor progression by secreting factors such as IL-10 and transforming growth factor β (TGF-β). This phenotype is frequently induced by hypoxia, which, through activation of the hypoxia-induced factor 1α (HIF-1α)/IL-1β pathway, increases the activity of pathways such as nuclear factor κB (NF-κB) and signal transducers and activators of transcription 3 (STAT3)—key regulators of inflammatory signaling and glioma cell survival [3,4,5,6]. Reactive oxygen species (ROS) generated during inflammation contribute to DNA damage and promote mutations, further enhancing the aggressive phenotype of GBM. These mechanisms activate apoptotic cascades in healthy cells while supporting the survival of cancer cells through metabolic adaptation.

Fluoride is one of the few environmental factors known to have pro-oxidative and pro-inflammatory effects [7]. Chronic exposure to fluoride causes dental and skeletal fluorosis [8], and its accumulation in soft tissues can damage multiple organs. In humans, plasma fluoride concentrations resulting from long-term ingestion of 1–10 ppm fluoride in drinking water typically range from 1 to 10 μM [9], although one study reports levels of 0.4–3.0 μM in healthy adults and up to 8–60 μM in fluorosis cases [10]. Notably, fluoride can cross the blood–brain barrier (BBB), raising concerns about neurotoxicity due to its ability to penetrate nervous tissue [11,12,13,14]. Fluoride can disrupt neurotransmitter metabolism, reduce glucose metabolism in neurons, and alter the profile of proteins involved in energy metabolism [15,16,17,18,19]. Moreover, fluoride exposure often triggers mechanisms associated with apoptosis and autophagy [3,4,20,21,22,23,24,25,26]. These molecular mechanisms are linked to cognitive and developmental deficits, including memory impairment, learning difficulties, and reduced IQ [11,27,28,29,30]. There is also evidence suggesting that fluoride compounds may increase the risk of dementia and ADHD [31,32], although some population studies have been criticized for methodological shortcomings [10,33,34,35].

Although direct epidemiological evidence linking fluoride exposure to glioblastoma (GBM) is limited, studies suggest that fluoride may impair neurological function, which is relevant given the brain origin of GBM. Further research is needed to clarify any relationship between fluoride exposure and GBM development or progression [2]. In our previous work, we found that treating U-87 cells with higher physiological NaF concentrations (0.1–10 μM) promoted a pro-invasive phenotype. Specifically, fluoride exposure increased U-87 cell migration across membranes and elevated secretion of matrix metalloproteinases MMP-2 and MMP-9. We also observed enhanced activation of invasiveness-related signaling pathways, including increased phosphorylation of AKT kinase and NF-κB. These findings suggest that fluoride exposure in vitro may render glioblastoma cells more aggressive and motile [36]. Fluoride has diverse effects on normal brain tissue, including inducing oxidative stress and neuroinflammation. Given the limited understanding of its effects on brain tumors, this study investigated the effects of physiologically relevant fluoride concentrations [9,10,37] on oxidative stress, antioxidant defenses, and inflammation-related signaling pathways in human U-87 glioblastoma cells. Understanding these effects may provide broader insight into the potential role of fluoride in modulating GBM progression and aggressiveness.

## 2. Materials and Methods

### 2.1. Cell Cultures

In all experiments, we used the human glioblastoma U-87 cell line (Sigma-Aldrich, Darmstadt, Germany). Cells were cultured in EMEM (EBSS; Sigma-Aldrich) supplemented with 10% fetal bovine serum (FBS), 2 mM glutamine, 1% non-essential amino acids, 1 mM sodium pyruvate, and antibiotics (100 IU/mL penicillin and 10 μg/mL streptomycin). Cultures were maintained at 37 °C in a 5% CO_2_ atmosphere with 95% humidity. To minimize variability from high passage number (which can affect cellular responses, protein expression, and transfection efficiency), the total number of passages during prolonged NaF exposure was strictly controlled [38].

We subjected U-87 cells to two NaF exposure regimens: short-term (3 days, one passage) and long-term (72 days, 24 passages). Sodium fluoride (NaF; Sigma-Aldrich) was added to culture media at final concentrations of 0.1, 1, 5, or 10 μM, chosen based on reported human serum levels. After each exposure period, cells were harvested and seeded into appropriate plates while maintaining the same NaF concentration. For subsequent analyses, cells were further incubated for an additional 12 or 48 h with the same NaF concentration. Depending on the assay, cells were detached either by trypsinization (0.25% trypsin) or by mechanical scraping. Trypsinization was used for routine detachment, whereas scraping was employed to preserve membrane proteins critical for certain assays [36].

We used strict aseptic techniques and regularly monitored cultures for contamination throughout the 72-day exposure. Passage number was limited to a predefined range, and mycoplasma testing was performed at the end of the experiment. Proliferation and cell viability were periodically assessed. Three independent culture replicates (in separate flasks) were performed. MTT assays confirmed that the NaF concentrations used did not induce cytotoxicity.

### 2.2. Intracellular Reactive Oxygen Species (ROS) Analysis

After short- and long-term exposure of U-87 cells to appropriate NaF concentrations (0.1–10 µM), cells were seeded into 6-well culture plates for both control and NaF-treated groups (0.1–10 µM). At specific time points (24, 48, and 72 h), cells were detached via trypsinization, washed twice with PBS, and suspended in a staining solution containing 0.1 µM 2′,7′-dichlorodihydrofluorescein diacetate (2′,7′-DCFH-DA, Sigma-Aldrich). Following a 30 min incubation (in the dark at room temperature), cells were washed twice with PBS and analyzed by flow cytometry (FACScan; Becton Dickinson, Franklin Lakes, NJ, USA; argon laser, λex = 488 nm). Fluorescence intensity in the green spectrum (FL-1, λem = 530 ± 15 nm) was measured for 10^4^ events per sample.

### 2.3. Antioxidant System Analysis

The activities of antioxidant enzymes (SOD and CAT) as well as total and reduced glutathione levels (GSH and GSSG) were assessed using commercial assay kits, following the manufacturers’ protocols. Cell lysates, prepared by sonication and multiple freeze–thaw cycles, were analyzed, and the results were normalized to 1 × 10^6^ cells. Spectrophotometric analyses were performed using an ASYS UVA 340 spectrophotometer (Biochrom, Cambridge, UK), with a minimum of three biological and technical replicates. The following parameters were quantified:

- Superoxide dismutase (SOD): Superoxide Dismutase Assay Kit (706002-96, CAYMAN, Ann Arbor, MI, USA)
- Catalase (CAT): Catalase Assay Kit (707002-96, CAYMAN)
- Glutathione (GSH and GSSG): Glutathione Assay Kit (703002-96, CAYMAN)

### 2.4. β-Catenin Analysis

Cells were fixed and permeabilized using the BD Cytofix/Cytoperm Fixation/Permeabilization Solution Kit (Becton Dickinson). The cells were then incubated with an Alexa Fluor 488–conjugated monoclonal antibody specific for β-catenin (Becton Dickinson) for 60 min in the dark at room temperature. After washing twice with Stain Buffer (Becton Dickinson), cells were analyzed by flow cytometry using a FACScan instrument (BD Biosciences, Franklin Lakes, NJ, USA) with an argon laser. Fluorescence intensity in the green spectrum (FL-1, λem = 530 ± 15 nm) was measured for 10^4^ events per sample.

### 2.5. Lysosome Analysis

Following short- and long-term exposure of U-87 cells to NaF (0.1–10 µM). Cells were seeded into 6-well culture plates for both control and NaF-treated groups (0.1–10 µM). Cells were then detached by trypsinization, washed twice with PBS, and incubated with LysoTracker Green DND-26 (100 nM, Invitrogen, Waltham, MA, USA) for 30 min in the dark at room temperature. Cells were washed twice with PBS and analyzed by flow cytometry (FACScan; Becton Dickinson, NJ, USA; argon laser, λex = 488 nm). Fluorescence intensity in the green spectrum (FL-1. λem = 530 ± 15 nm) was measured for 10^4^ events per sample.

### 2.6. Enzyme-Linked Immunosorbent Assay (ELISA)

Quantitative analysis of target proteins was performed using sandwich ELISA. This method involves placing a solution containing the sample on a plate pre-coated with antibodies specific to the target protein. After incubation, unbound components are removed by washing, and a secondary antibody conjugated with horseradish peroxidase (HRP) is added. Following substrate addition, a colorimetric reaction occurs, and absorbance is measured spectrophotometrically. The intensity of the color corresponds to the amount of protein bound during the initial stage, with concentrations calculated based on a standard curve of known protein concentrations.

Commercial ELISA kits were used according to the manufacturer’s instructions. Cell lysates (prepared via sonication/freezing-thawing) and culture medium samples were analyzed, with results normalized and expressed per 10^6^ cells in each experimental group. Measurements were performed using an ASYS UVA 340 spectrophotometer, with at least three biological and technical replicates. The following proteins were analyzed:

- Tumor necrosis factor α (TNF-α): Human TNF-α ELISA Kit (E0133h, Wuhan EIAab Elisas, Wuhan, China), measured in both medium and cell lysates.
- Interleukin 1α (IL-1α): Human IL1A/Interleukin-1alpha ELISA Kit (E0071h, Wuhan EIAab Elisas), measured in both medium and cell lysates.
- Interleukin 1β (IL-1β): Human IL1B/Interleukin-1beta ELISA Kit (E0563h, Wuhan EIAab Elisas), measured in both medium and cell lysates.
- Interleukin 4 (IL-4): Human IL4/Interleukin-4 ELISA Kit (E0077h, Wuhan EIAab Elisas), measured in both medium and cell lysates.
- Interleukin 10 (IL-10): Human IL10/Interleukin-10 ELISA Kit (E0056h, Wuhan EIAab Elisas), measured in both medium and cell lysates.
- Interferon γ (IFN-γ): Human IFNG/Interferon gamma ELISA Kit (E0049h, Wuhan EIAab Elisas), measured in both medium and cell lysates.
- Interleukin 6 (IL-6): Human IL6/Interleukin-6 ELISA Kit (E0079h, Wuhan EIAab Elisas), measured in both medium and cell lysates.
- Thromboxane B2 (TXB2): Thromboxane B2 Express ELISA Kit—Monoclonal (10004023-96, CAYMAN), measured in cell lysates.
- Prostaglandin E2 (PGE2): Prostaglandin E2 ELISA Kit—Monoclonal (514010-96, CAYMAN), measured in both medium and cell lysates.

### 2.7. Statistical Analysis

Statistical analysis was performed using Statistica 13.3 (TIBCO). Data are presented as the mean ± standard deviation (SD) of three independent biological replicates (*n* = 9). Because the data were not normally distributed, nonparametric tests were used. Differences between short- and long-term exposure for each NaF concentration were assessed using the Mann–Whitney U test (# *p* ≤ 0.05; ## *p* ≤ 0.01). Within-group differences between NaF concentrations and controls were evaluated using the Wilcoxon test (* *p* ≤ 0.05; ** *p* ≤ 0.01; *** *p* ≤ 0.001). No correction for multiple testing was applied due to the exploratory nature of the study and the focus on individual comparisons. However, potential type I errors should be considered when interpreting the results.

## 3. Results

**Figure 1:**
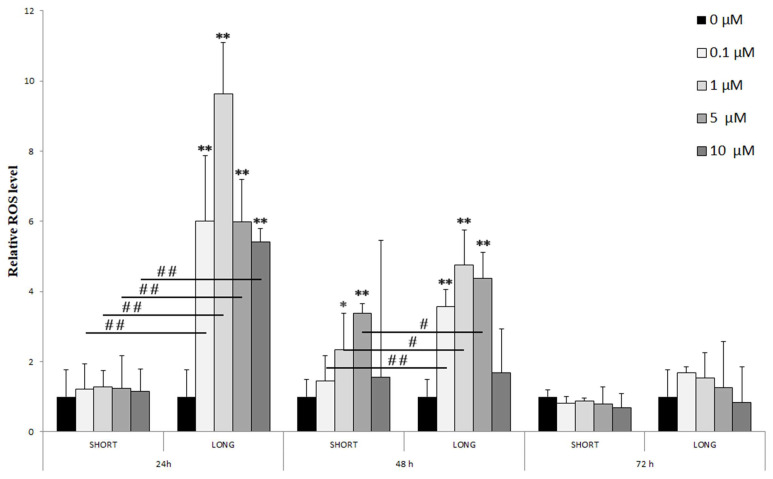
Relative levels of reactive oxygen species (ROS) in control cells and experimental systems (0.1–10 µM NaF, t = 24, 48, and 72 h, short- and long-term exposure). Data represent arithmetic means ± SD; analysis of differences between exposure times: Mann–Whitney U test; # *p* ≤ 0.05, ## *p* ≤ 0.01; analysis of differences within a single exposure time: Wilcoxon test; * *p* ≤ 0.05, ** *p* ≤ 0.01. Experiments were performed in 3 independent technical and biological replicates (*n* = 9).

Flow cytometry analysis showed that NaF concentrations influenced reactive oxygen species (ROS) production in U-87 cells (Figure 1).

Figure 1 shows the relative ROS levels in U-87 glioma cells exposed to NaF at concentrations of 0.1, 1, 5, and 10 µM under different exposure durations (24, 48, and 72 h) and modes (SHORT—short-term; LONG—long-term).

- 24 h: A significant increase in ROS levels was observed only with long-term (LONG) exposure to all tested NaF doses (0.1–10 µM), reaching a maximum at 1 µM (** *p* < 0.01). Significant differences between doses were marked (# *p* < 0.01), indicating a dose-dependency of the effect.
- 48 h: The increase in ROS levels was visible with both short (SHORT) and long (LONG) exposures, although more pronounced with LONG. The effect was again greatest for 1 µM NaF (** *p* < 0.01 LONG). Differences between concentration groups were also significant (# *p* < 0.05, ## *p* < 0.01).
- 72 h: After 72 h, ROS levels were significantly lower than at 24 and 48 h, with no statistically significant differences from the control, which may suggest cell adaptation to long-term exposure to NaF.

Sodium fluoride significantly induced ROS production in U-87 cells, especially after longer exposures (24–48 h). This effect decreased over time, which may indicate the activation of adaptive antioxidant mechanisms.

**Figure 2:**
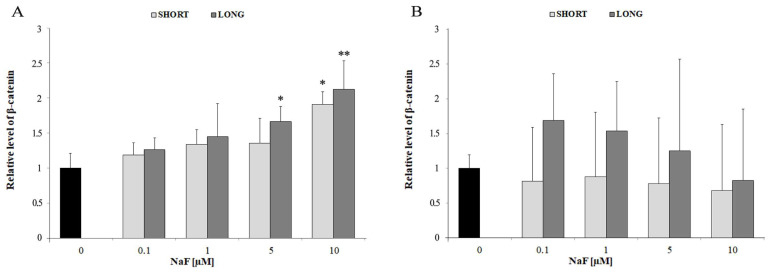
The effect of different concentrations of NaF (0.1–10 µM) and exposure time (3 days—SHORT, 72 days—LONG) on the levels of β-catenin in glioma cells (**A**) and post-culture medium (**B**) exposed to NaF, indicating their secretion into the extracellular space. Data represent arithmetic means and standard deviations; analysis of differences between exposure times: Mann–Whitney U test; analysis of differences within groups: Wilcoxon test; * *p* ≤ 0.05, ** *p* ≤ 0.01. Three biological and technical replicates were performed (*n* = 9).

A significant increase in β-catenin levels was observed at NaF doses of 5 and 10 µM for both short-term and long-term exposures (* *p* < 0.05 and ** *p* < 0.01, respectively). The increase was stronger after long-term exposure, suggesting enhanced Wnt pathway activation under chronic NaF treatment (Figure 2A). No significant changes were observed in β-catenin levels in the culture medium, indicating that Wnt pathway activation remains mainly intracellular and does not lead to substantial extracellular β-catenin accumulation (Figure 2B). These results indicate that sodium fluoride activates the Wnt pathway in glioma cells, as evidenced by increased intracellular β-catenin levels, particularly at higher concentrations and longer exposure times.

**Figure 3:**
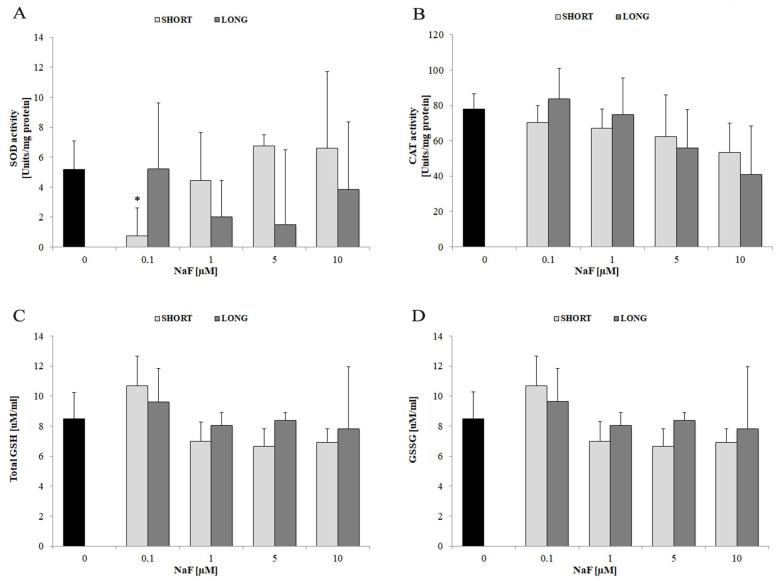
Antioxidant enzyme activity (SOD, CAT) and glutathione levels (GSH, GSSG). The effect of different concentrations of NaF (0.1–10 µM) and exposure time (3 days—SHORT, 72 days—LONG) on the levels of SOD activity (**A**), CAT activity (**B**), total GSH (**C**) and GSSG (**D**) in glioma cells exposed to NaF, indicating their secretion into the extracellular space. Data represent arithmetic means and standard deviations; analysis of differences between exposure times: Mann–Whitney U test; analysis of differences within groups: Wilcoxon test; * *p* ≤ 0.05, Three biological and technical replicates were performed (*n* = 9).

A significant decrease in SOD activity was observed with short exposure to 0.1 µM NaF (* *p* < 0.05). No significant differences were observed for the remaining doses and exposure times, suggesting only a transient decrease in the activity of this enzyme at a low dose and short time (Figure 3A). At the same time, a clear downward trend in catalase activity was noted with increasing NaF concentration (especially at 5–10 µM), although this did not reach statistical significance (Figure 3B). The levels of total glutathione—total GSH (Figure 3C) and oxidized form of glutathione—GSSG (Figure 3D) did not differ statistically significantly from the control, although a tendency for a slight decrease with increasing NaF dose can be observed (especially for GSH at 5–10 µM).

Exposure of glioma cells to sodium fluoride leads to moderate disturbances in the antioxidant system (transient decrease in SOD activity at low dose and tendency to decrease in CAT); however, the overall glutathione level remains relatively stable. This suggests partial compensation of oxidative stress by glioma cells during exposure to NaF.

**Figure 4:**
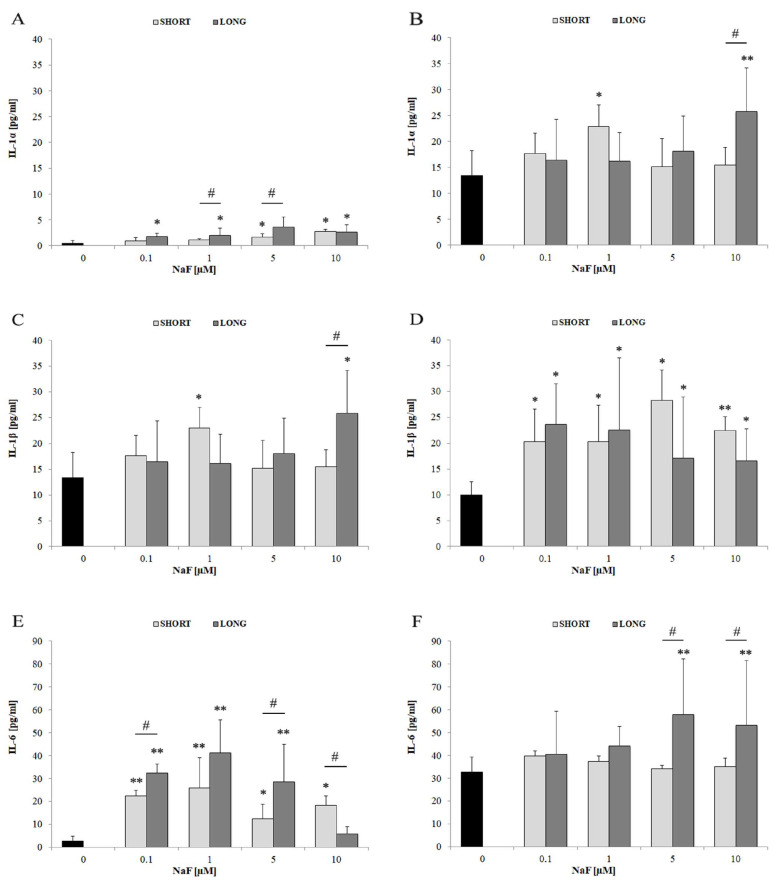
The effect of different concentrations of NaF (0.1–10 µM) and exposure time (3 days—SHORT, 72 days—LONG) on the levels of three pro-inflammatory cytokines: IL-1α (**A**,**B**), IL-1β (**C**,**D**) and IL-6 (**E**,**F**). The graphs on the left (**A**,**C**,**E**) show the level of cytokines in the U-87 cell lysate, while those on the right (**B**,**D**,**F**)—in the post-culture medium, indicating their secretion into the extracellular space. Data represent arithmetic means and standard deviations; analysis of differences between exposure times: Mann–Whitney U test; # *p* ≤ 0.05; analysis of differences within groups: Wilcoxon test; * *p* ≤ 0.05, ** *p* ≤ 0.01. Three biological and technical replicates were performed (*n* = 9).

The IL-1α level increased at all NaF concentrations (* *p* < 0.05), with a significant difference between SHORT and LONG for 1 and 5 µM (# *p* < 0.05), although the overall level remained low (Figure 4A). IL-1α secretion was more strongly induced at 10 µM, especially after longer exposure (** *p* < 0.01; # *p* < 0.05) (Figure 4B). IL-1β level in U-87 cells increased only moderately and irregularly, with a statistically significant increase only at 1 µM (* *p* < 0.05), without a clear dose-dependent trend (Figure 4C). In contrast, IL-1β secretion was more pronounced—a significant increase at all concentrations (* *p* < 0.05; ** *p* < 0.01), especially with long-term exposure, which indicates active production and release of this cytokine (Figure 4D). Moreover, a significant increase in IL-6 levels was observed in cells at all NaF concentrations, especially after long-term exposure (** *p* < 0.01), with a clear upward trend, dose- and time-dependent (# *p* < 0.05) (Figure 4E). IL-6 levels in the medium also increased, with the highest values for 5 and 10 µM NaF (** *p* < 0.01), and the differences between SHORT and LONG were significant for the highest doses (Figure 4F).

IL-6 shows the strongest response among the analyzed cytokines—both in cells and in the medium. IL-1α and IL-1β are secreted more than stored intracellularly, suggesting their rapid release after activation. Long-term exposure (LONG) enhances the activity of inflammation-related pathways for all analyzed interleukins.

**Figure 5:**
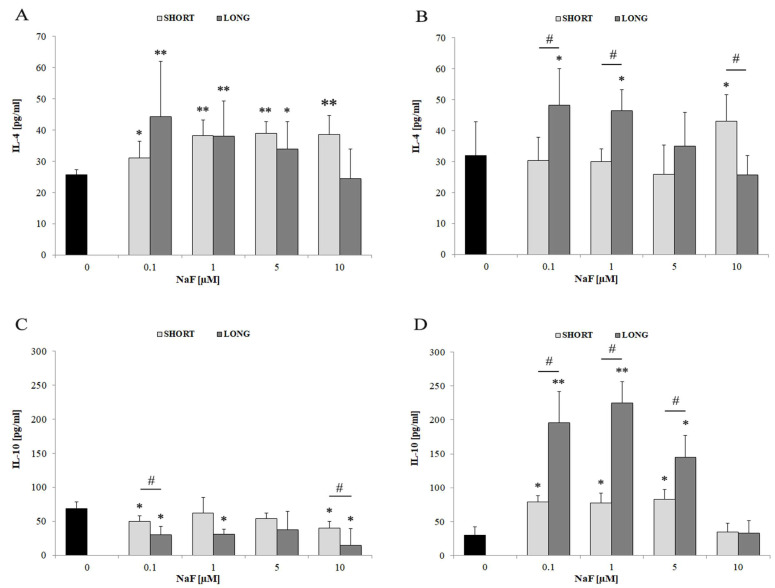
The effect of different concentrations of NaF (0.1–10 µM) and exposure time (3 days—SHORT, 72 days—LONG) on the levels of two anti-inflammatory cytokines: IL-4 (**A**,**B**) and IL-10 (**C**,**D**). The graphs on the left (**A**,**C**) show the level of cytokines in the U-87 cell lysate, while those on the right (**B**,**D**)—in the post-culture medium, indicating their secretion into the extracellular space. Data represent arithmetic means and standard deviations; analysis of differences between exposure times: Mann–Whitney U test; # *p* ≤ 0.05,; analysis of differences within groups: Wilcoxon test; * *p* ≤ 0.05, ** *p* ≤ 0.01. Three biological and technical replicates were performed (*n* = 9).

Exposure to all tested concentrations of NaF led to a significant increase in IL-4 levels compared to the control (* *p* < 0.05, ** *p* < 0.01) in cell lysate, with the greatest effect observed at 0.1 and 1 µM. No significant differences were observed between exposure times (Figure 5A). A significant increase in IL-4 levels was also demonstrated in the post-culture medium at 0.1–1 µM NaF (* *p* < 0.05), but this effect was stronger after long-term exposure (# *p* < 0.05), which may suggest a time-dependent secretion of this cytokine (Figure 5B). In contrast to IL-4, the level of IL-10 in cells was significantly reduced after exposure to NaF (* *p* < 0.05), especially at lower concentrations (0.1–1 µM), with significant differences between SHORT and LONG (# *p* < 0.05), indicating a decrease in the intracellular content of IL-10 (Figure 5C). On the other hand, the level of IL-10 in the medium increased significantly, especially after long-term exposure to 0.1–5 µM NaF (** *p* < 0.01), which may indicate active secretion of IL-10 despite the reduced intracellular level (Figure 5D).

NaF induces the production and secretion of IL-4 and IL-10 in a concentration- and time-dependent manner. In the case of IL-10, a discrepancy between intracellular levels and secretion was observed, which may indicate a rapid release of this cytokine in response to the stress associated with NaF exposure.

**Figure 6:**
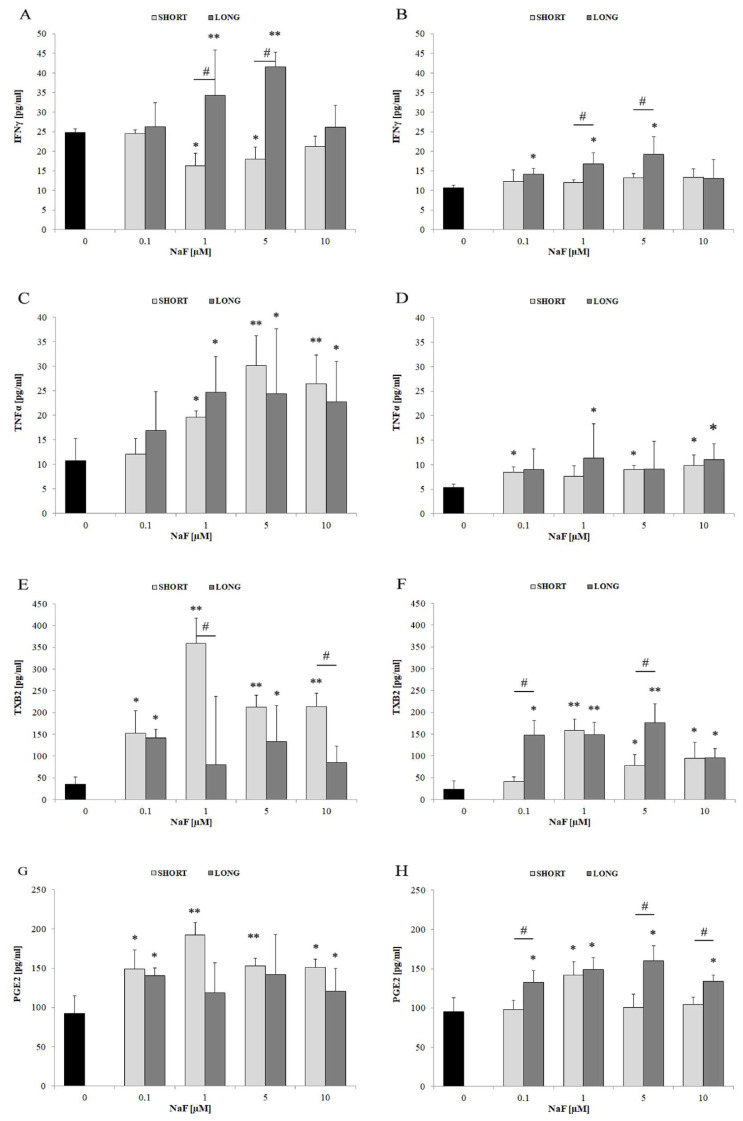
The effect of different concentrations of NaF (0.1–10 µM) and exposure time (3 days—SHORT, 72 days—LONG) on the levels of pro-inflammatory factors: IFN-γ (**A**,**B**), TNF-α (**C**,**D**), TXB2 (**E**,**F**) and PGE2 (**G**,**H**). The graphs on the left (**A**,**C**,**E**,**G**) show the level of factors in the U-87 cell lysate, while those on the right (**B**,**D**,**F**,**H**)—in the post-culture medium, indicating their secretion into the extracellular space. Data represent arithmetic means and standard deviations; analysis of differences between exposure times: Mann–Whitney U test; # *p* ≤ 0.05,; analysis of differences within groups: Wilcoxon test; * *p* ≤ 0.05, ** *p* ≤ 0.01. Three biological and technical replicates were performed (*n* = 9).

In the cell lysate (Figure 6A), a clear increase in the level of IFN-γ was observed in response to NaF, especially at concentrations of 1 and 5 µM. The effect was stronger after long-term exposure (LONG) (** *p* < 0.01 vs. control; # *p* < 0.05 vs. SHORT). In the post-culture medium (Figure 6B), the level of IFN-γ also increased significantly (* *p* < 0.05) for 1 and 5 µM, but the magnitude of changes was smaller than in cells, suggesting moderate secretion of this mediator. Additionally, in the lysate (Figure 6C), a significant increase in TNF-α was found at 1–10 µM NaF, stronger after longer exposure (** *p* < 0.01). In the medium (Figure 6D), the changes were more subdued, although still statistically significant (* *p* < 0.05), which may suggest limited secretion of TNF-α or its rapid extracellular degradation. TXB2 showed the strongest response among all mediators tested. In the lysate (Figure 6E), TXB2 levels increased significantly at all NaF concentrations, with a maximum at 1 µM (** *p* < 0.01; # *p* < 0.05 between exposure times). In the medium (Figure 6F), a similar trend was observed—a significant increase already at 0.1 µM (* *p* < 0.05), with the effect dependent on both concentration and time (# *p* < 0.05). The strong presence of TXB2 in the medium indicates active secretion by U-87 cells. In the cell lysate (Figure 6G), the PGE2 level was significantly higher after exposure to NaF in a concentration- and time-dependent manner (maximum at 1–5 µM; * *p* < 0.05; ** *p* < 0.01), while in the post-culture medium (Figure 6H), a significant increase was also noted, especially after long-term exposure (# *p* < 0.05), confirming the active secretion of PGE2 as a response to NaF-induced stress.

NaF induces an inflammatory response in U-87 cells, as evidenced by an increase in the levels of IFN-γ, TNF-α, TXB2, and PGE2 both intracellularly and in the culture medium. The strongest effect is observed for TXB2 and PGE2, which may indicate a specific involvement of the eicosanoid axis in the response to NaF. Cytokine levels are generally higher in the cell lysate than in the medium, suggesting intracellular accumulation of some mediators or limited secretion. The time of exposure to NaF significantly affects the inflammatory response—long-term action of the compound intensifies the production of most of the cytokines tested.

## 4. Discussion

### 4.1. Oxidative Stress and Antioxidant Enzymes

In this study, we demonstrated that NaF altered the levels of reactive oxygen species (ROS) in U-87 cells. For cells exposed to short-term fluoride treatment, increased ROS levels were observed at 48 h. In contrast, in cells subjected to long-term exposure, a significant rise in ROS occurred as early as 24 h and persisted until 48 h. Currently, there is no available literature on the effects of NaF on ROS production in GBM cells, but several studies suggest that fluoride can promote ROS generation in brain tissues [39,40,41].

The increased ROS levels imply elevated redox stress in U-87 glioma cells under NaF exposure. As neurological damage following fluoride exposure has been linked to oxidative stress [6], we further analyzed the effect of NaF on the enzymatic antioxidant system in U-87 cells. Lysate analysis revealed changes in SOD and CAT concentrations in cells incubated with NaF.

The literature on the effects of fluoride compounds on the levels and activity of antioxidant enzymes is limited, especially in the context of central nervous system tumor cells. In one of the few reports analyzing the effects of NaF on antioxidant enzymes in various rat brain structures, exposure to NaF (50 mg/L) was shown to disrupt NADPH oxidase 4 (NOX4), CAT, SOD, GPx, GSH, and total antioxidant capacity (TAC) in the cerebellum, prefrontal cortex, hippocampus, and striatum. Significant changes were observed in the activity of all tested antioxidant enzymes and a decrease in TAC levels in the analyzed brain regions. It was suggested that NOX4 induction and reduced antioxidant activity in central nervous system (CNS) cells might be the primary mechanisms of fluoride neurotoxicity [21]. Similar in vivo findings were presented by another group, which demonstrated that rats exposed to high fluoride doses exhibited impaired learning and memory abilities, reduced SOD activity, and increased ROS and malondialdehyde (MDA) levels in various brain regions compared to the control group [42]. Additionally, excessive activation of antioxidant enzymes such as CAT and a pronounced decrease in SOD and glutathione transferase (GST) activities were observed in neural cells of Drosophila melanogaster following fluoride exposure [43]. In another rat study, where animals received water containing 0, 15, or 50 mg/L F (as NaF) for 20 or 60 days, SOD, GPx, and CAT levels were significantly reduced during short-term exposure regardless of dose. However, during long-term exposure, enzyme levels in the serum increased or decreased depending on the duration. Short-term exposure was characterized by lower ROS levels, whereas long-term exposure showed variability in ROS levels. Collectively, this suggests an adaptive response of the organism to effectively remove generated ROS [44]. Another study revealed that male rats supplemented with NaF in drinking water exhibited disruptions (reductions) in SOD, CAT, GPx, and GSH levels in testicular tissue [45]. These results were consistent with previous findings, where SOD and CAT activities in the testes and epididymis of rats were significantly reduced following exposure to 50 or 100 mg NaF. Moreover, the mRNA expression of SOD1 and CAT was significantly decreased in the testes of rats exposed to 100 mg NaF/L in drinking water, while in the epididymis, mRNA expression of SOD1 and CAT was reduced across all fluoride-exposed groups [46]. Another study demonstrated that rats exposed to NaF exhibited significant increases in serum urea, creatinine, uric acid, nitric oxide, and TNF-α, accompanied by elevated lipid peroxidation in the kidneys. These changes correlated with reduced SOD and GSH levels in the serum [47]. Similar findings were reported in the liver of chickens supplemented with NaF. The results showed that fluoride doses of 400 mg/kg body weight reduced CAT levels, 800 mg/kg reduced SOD levels, and 600 mg/kg decreased the total antioxidant capacity in liver tissue [48]. However, another study on chickens exposed to various NaF concentrations for 6 h did not show any significant differences compared to the control group in the expression of mRNA or the immunolocalization of CAT, SOD1, and SOD2 in their gonads [49].

The presented analysis of U-87 cell lysates exposed to NaF, both short- and long-term, did not show statistically significant changes in the GSH/GSSG ratio. However, a tendency for an initial increase followed by a gradual decrease in GSH and GSSG levels was observed with increasing fluoride concentration.

Since glutathione is the primary antioxidant involved, alongside antioxidant enzymes, in reducing free radicals in cells, it is possible that fluoride exposure at concentrations causing a significant increase in ROS was partially buffered by GSH [50]. The reduced GSH/GSSG levels may also result from decreased ATP bioavailability due to inhibition of the Krebs cycle and the respiratory chain, as glutathione synthesis involves two ATP-dependent enzymatic reactions [51]. This is consistent with previous literature data suggesting that fluoride’s effect on cellular energy metabolism depends on both exposure time and concentration. Thus, it can be hypothesized that the antioxidant system in U-87 glioma cells attempts to mitigate ROS levels induced by NaF exposure through GSH/GSSG conversion. Similar findings were observed in studies analyzing the effects of long-term fluoride exposure on rat hepatocytes, where oxidative stress disrupted cellular homeostasis [52]. This is consistent with the results presented in this study, suggesting that NaF exposure can also disturb the redox homeostasis in U-87 cells.

It should be emphasized that only the lowest NaF concentrations assessed in this study (0.1–5 µM) are comparable to fluoride levels observed in the plasma of individuals living in areas with endemic fluoride contamination. However, the effects of fluoride compounds on the body depend not only on dose but also on exposure duration [53]. Araújo et al. [44] reported possible disruptions in mitochondrial energy metabolism in rats treated with water containing 50 μg/mL F for 15 days. However, when intoxication was prolonged to 60 days, the changes became less noticeable, suggesting the organism’s potential adaptive capacity to mitigate the adverse effects of fluoride.

The presented results confirm disturbances in redox homeostasis in U-87 glioma cells under the influence of NaF, manifested by increased ROS production and modulation of SOD and CAT activity. Although this study provides a novel analysis of the effects of fluoride in a glioma model, the observed variability of the enzymatic response compared to other tissues indicates the complexity of the mechanisms of fluoride toxicity, requiring further glioma-specific analyses. The observed tendency to reduce glutathione levels at higher NaF concentrations, preceded by initial stress compensation, provides valuable information on the adaptive limits of the antioxidant system.

### 4.2. The Role of Fluorides in Regulating β-Catenin Levels

Based on the obtained results, it appears that NaF can influence cytosolic β-catenin levels in U-87 cells. It was also shown that intracellular β-catenin levels increased with rising NaF concentrations in U-87 cells. The impact of fluoride on Wnt signaling activation in cancer cells is not well understood. However, several studies have highlighted the role of fluoride in Wnt/β-catenin signaling in healthy tissues. One study demonstrated a potential mechanism of abnormal osteoblast activation induced by fluoride ions, suggesting that β-catenin is a key mediator of osteoblast survival/differentiation and could serve as a therapeutic target in skeletal fluorosis [54]. Similar conclusions were drawn by another research group [55], where an increase in the Wnt antagonist Dickkopf Wnt Signaling (Dkk-1) was also observed. Furthermore, another study indicates that prolonged exposure to elevated fluoride levels can reduce the levels of physiological inhibitors of Wnt/β-catenin pathway activation [56]. This relationship is further supported by research showing that fluoride and arsenic exposure gradually increased Wnt/β-catenin pathway activation, while the Dkk-1 content significantly decreased [57]. Conversely, fluorides were shown to increase IL-6, TNF-α, and ROS production, promoting inflammatory signaling and oxidative stress while simultaneously inhibiting canonical Wnt signaling and stimulating NF-κB pathway activation in BV2 microglial cells [6]. In a study by Luo et al., it was demonstrated for the first time that activation of the Wnt9a/β-catenin/CyclinD1 pathway in osteoblasts occurred under fluoride exposure [58]. Other studies revealed that NaF activated both canonical and non-canonical Wnt signaling pathways in ameloblast cell lines in vitro. The levels of Gsk-3β and axin-1 were significantly reduced following stimulation with 1.5 mM NaF, while Dvl3 levels increased. The canonical and non-canonical Wnt family proteins Wnt3a and Wnt5a were significantly upregulated under NaF treatment [59]. It was also confirmed that both the Wnt and Rho signaling pathways were enhanced by 1.5 mM NaF solution [60]. Moreover, excessive fluoride consumption (5–50 ppm F^−^) by rats led to calpain-1 stimulation (a proteolytic enzyme), which was accompanied by a significant decrease in cytoplasmic RhoA levels in hippocampal cells and a marked increase in its expression in cell membranes [61].

If the increase in intracellular β-catenin levels upon NaF treatment was associated with the activation of the Wnt pathway, one could also consider the role of fluoride in exacerbating multidrug resistance and avoiding apoptosis in glioma cells. Some studies indicate that the activated Wnt/β-catenin signaling pathway induces autophagy-dependent resistance to temozolomide (TMZ) in human glioma [62], but there is currently no literature data indicating a possible association of fluoride effects with the development of TMZ resistance in glioma cells. However, it should be noted that studies on the effects of fluoride compounds on these processes in the brain remain limited. An intriguing clue comes from the data presented in this study regarding lysosomal integrity. These results demonstrated a significant increase in the accumulation of a lysosome-specific marker in groups subjected to prolonged NaF exposure, which indirectly indicates an increase in the number of these organelles. It is well known that lysosomes fuse with double-membrane-bound autophagosomes during the autophagic process, forming single-membrane autolysosomes. This fusion allows the digestion of autophagosome content by lysosomal enzymes, such as hydrolases. The autophagic process requires the generation of a reserve of lysosomes, as more than one lysosome fuses with a single autophagosome [63].

While the results presented here do not permit such bold conclusions, we have proposed certain hypotheses that, in our opinion (particularly concerning β-catenin levels), should serve as encouragement for further investigation into the role of fluoride in this signaling pathway.

### 4.3. The Role of Fluorides in Regulating Inflammatory Cytokines

This study analyzed the concentrations of cytokines that are critical to inflammatory signaling, tumor progression, and invasiveness of GBM cells. It was demonstrated that NaF can influence the levels of the pro-inflammatory cytokines IL-6, IL-1α, IL-1β and the anti-inflammatory cytokines IL-4 and IL-10, both in the culture medium and within the cells. The duration of NaF exposure (SHORT vs. LONG) had a clear effect on cytokine profiles, highlighting the kinetic differences between pro- and anti-inflammatory responses. In general, short-term exposure resulted in a rapid release of pro-inflammatory cytokines, whereas prolonged exposure led to a more pronounced increase in anti-inflammatory cytokines, particularly IL-10. For example, IL-1α, IL-1β, and IL-6 were already elevated in SHORT treatment, indicating that these are early response cytokines. Their levels either remained elevated or increased further in LONG exposure at specific NaF concentrations (suggesting ongoing signaling or accumulation). IL-10, however, showed a clear time-dependent pattern, with only minor changes in the short term but a dramatic increase after longer exposure (especially seen at 1 μM NaF, where IL-10 in LONG exposure was many times higher than in SHORT). This delayed induction may be related to the fact that IL-10 is a late response gene, requiring continuous activation signals or secondary feedback loops (e.g., IL-1β/NF-κB signaling for several hours) to achieve high expression [64]. IL-4 also tended to be more elevated with long exposures, indicating that whatever mechanism drives IL-4 production, it likely also requires prolonged stimulation. At longer time points, the cultured cells may be in a state of increased cytokine turnover—both inflammatory and anti-inflammatory—which may indicate an attempt to restore homeostasis following the initial NaF insult.

#### 4.3.1. IL-6

For IL-6, a slight increase in protein levels was observed in the culture medium after short-term exposure, whereas a pronounced increase was noted after long-term exposure, especially at higher NaF concentrations. Results from cellular lysate analysis showed a significantly higher level of IL-6 regardless of NaF concentration and exposure duration. IL-6 is a factor involved in glioblastoma malignancy, promoting processes such as renewal, invasion, and angiogenesis [65]. Additionally, IL-6 drives three key signaling pathways associated with gliogenesis: p42/p44-MAPK, PI3K/AKT, and JAK-STAT3. The results obtained indicate that the elevated IL-6 levels in cells exposed to NaF correlate with increased protein kinase B (AKT) kinase activation, as shown in our previous studies [36] and align with other literature findings [66]. Furthermore, increased NF-κB activity—a known regulator of IL-6 expression—supports the notion that NaF may stimulate metabolic pathways linked to GBM cell progression and invasiveness [36].

#### 4.3.2. IL-1α and IL-1β

For both IL-1α and IL-1β isoforms, a significant increase in IL-1 protein levels was observed in both the culture medium and cellular lysates. Additionally, both short- and long-term NaF exposure stimulated the production and secretion of IL-1α and IL-1β, with some differences at certain concentrations. The secretion of IL-1α by cancer cells is associated with constitutive NF-κB activation, which drives the expression of downstream target genes involved in progression, metastasis, and angiogenesis [67]. Furthermore, NF-κB can be additionally activated by an IL-1α feedback loop, leading to increased levels of hyaluronic acid in the GBM microenvironment, which promotes tumor invasion [68]. Moreover, elevated IL-1β levels observed in various GBM cell lines, including CCF3 and U-87 [69], U373MG [70] and in human GBM tumor samples [71] were associated with the activation of the NF-κB and mitogen-activated protein (MAPK) signaling cascades, enhancing tumor cell invasiveness. This aligns with the elevated NF-κB activity demonstrated in our previous studies [36].

#### 4.3.3. IL-4 and IL-10

NaF exposure also increased the levels of the anti-inflammatory cytokines IL-4 and IL-10 in cultured U-87 cells. Both IL-4 and IL-10 increased in parallel with pro-inflammatory cytokines, suggesting that NaF triggers a complex immunoregulatory response rather than a unidirectional increase in inflammatory signaling. One explanation could be the activation of intrinsic negative feedback mechanisms. Some cells (such as microglia) increase the expression of anti-inflammatory mediators to counteract the excessive inflammation.

On the other hand, elevated IL-4 levels may suggest that fluoride may have a potentially inhibitory effect on glioblastoma progression and invasiveness. Most studies indicate that IL-4 correlates with GBM tumor regression, although the mechanism remains unclear [72]. However, the potential role of IL-4 in the formation of alternatively activated M2 macrophages [73] could play a significant role in GBM progression and invasion. The increase in IL-10 levels in the culture medium may also represent an adverse effect of NaF on GBM cells. High levels of this interleukin are associated with poor prognosis in GBM patients [74]. As early as the 1990s, it was observed that IL-10 promotes the infiltration of surrounding tissues by GBM cells [75]. Recent studies have shown that IL-10 significantly enhances the invasive potential and migration of U-87 glioblastoma cells in vitro [76]. Combined with IL-10′s ability to induce the secretion of MMP-2 and -9 by macrophages [77], the results presented in this study provide new context for the role of fluoride compounds in regulating inflammatory signaling in GBM.

### 4.4. TNFα

The analysis of the culture medium and cellular lysates revealed that NaF caused a slight increase in TNFα concentration in the medium (practically across all experimental systems) and a substantial increase in cellular TNFα levels (in all systems, regardless of exposure time). Additionally, TNFα levels in cells increased with rising NaF concentrations.

Previous studies suggest that TNFα is involved in reduced macrophage infiltration in histopathological studies, indicating that TNFα may have a suppressive role alongside its tumor-promoting abilities [78]. Unfortunately, in the case of GBM, high TNFα levels can activate the JNK-Axl-ERK signaling pathway, which mediates resistance to epidermal growth factor receptor (EGFR) inhibition [79]. TNFα has also been shown to induce angiogenesis in malignant glioblastoma cells [80], highlighting its critical role in tumor progression in GBM.

In summary, there is currently no direct evidence linking fluoride exposure to the production and secretion of IL-6, IL-1α, IL-1β, TNF-α, IL-4, and IL-10 in GBM cells. However, isolated studies indicate that fluoride exposure is associated with inflammation in various tissues, such as gonads and the eye, resulting in increased pro-inflammatory cytokines, including IL-6 [81,82]. The role of pro-inflammatory cytokines in fluoride toxicity has been studied in Hepa1-6 cells cultured in medium containing fluoride concentrations of 0, 0.5, 1.0, 1.5, 2.0, 3.0, 4.0, and 5.0 mmol/L. IL-6, IL-1β, and TNFα concentrations in the supernatant were significantly reduced compared to controls in groups exposed to NaF concentrations of 1 mmol/L or higher. In contrast, fluoride concentrations below 0.5 mmol/L increased cytokine levels in the supernatant [83]. Similar observations were made in a mouse model, where liver inflammation was associated with MAPK and NF-κB pathway activation and increased levels of IL-1β, IL-6, IL-8, cyclooxygenase 2 (COX-2), and monocyte chemotactic protein-1(MCP-1) in tissue [22]. Another study showed that fluoride-induced synthesis of IL-6 and IL-8 occurred in human A549 lung epithelial cells [84]. Additionally, increased levels of IL-6, IL-8, TNFα, and IL-1β induced by NaF through NF-κB pathway activation and a decrease in IL-4 and IL-10 were observed in mouse kidneys [85]. In cervical cancer HeLa cells cultured with fluoride concentrations of 1–50 mg/L for 48 h, there was a strong increase in IL-1β, IL-6, and TNFα levels in the supernatant at the lowest concentration (1 mg/L), followed by a significant decrease at higher concentrations. This is consistent with the present findings, as the concentrations used in this study were at least an order of magnitude lower. Finally, in BV-2 human microglial cells, it was shown that low fluoride concentrations caused a significant increase in IL-1β levels, higher concentrations increased TNFα synthesis, and very high concentrations reduced both cytokines to below control levels [86].

### 4.5. The Role of Fluorides in Modulating Prostanoid Metabolism

The results obtained from analyses of the culture medium and lysates of U-87 cells exposed to NaF indicate a significant increase in PGE2 levels in both the medium and in the cells. Additionally, an increase in TXB2 levels was observed in both the medium and cells across all NaF-treated systems. Currently, there is no literature directly indicating or suggesting that fluoride may influence prostanoid metabolism in GBM cells. However, several pieces of evidence from other model systems suggest possible connections. It is known that fluoride exposure, through increased production of inflammatory cytokines, may lead to enhanced activity of transcription factors and enzymes involved in inflammatory signaling (e.g., activation of NF-κB and COX-2) and subsequent production of prostanoids, including PGE2 and TXB2, in human THP-1 macrophages [87,88]. Furthermore, the addition of NaF to purified platelets causes a dose-dependent and transient increase in intracellular free calcium concentration ([Ca++]) and elevated TXB2 synthesis [89]. High serum NaF levels in rabbits have also been shown to correlate with increased serum TXB2 concentrations [90]. Fluoride ions have also been found to influence the activity of antioxidant enzymes, COX-2 protein expression, and PGE2 synthesis in the livers of NaF-supplemented rats [91]. This is supported by earlier studies showing a significant increase in COX-2 activity and PGE2 levels in human A549 lung epithelial cells in response to NaF exposure [92]. Further findings demonstrated that NaF doses exceeding 12 mg/kg body weight induced histopathological changes in the kidneys, activated the NF-κB signaling pathway, and led to increased PGE2 synthesis, heightened activity and levels of inducible nitric oxide synthase (iNOS) and COX-2, as well as elevated TNFα, IL-1β, and IL-6 concentrations [85]. Similar results were obtained in studies on human gingival fibroblasts [93]. It has also been shown that fluoride can, in some cases, activate the IGF-1 signaling pathway and significantly increase insulin like growth factor 1 (IGF-1) levels in serum [94]. Although the molecular mechanism of this process is unknown, it is established that fluoride at very low concentrations (1–10 µM NaF) stimulates PGE2 synthesis [87]. Increased PGE2 may promote IGF-1 synthesis via the cyclic adenosine-3′,5′-monophosphate (AMP)/protein kinase type A (PKA) pathway [89].

The results presented in this study appear consistent with the literature, which suggests a stimulatory role for NaF in the synthesis of prostanoids (PGE2 and TXB2) and implicates the activation of inflammation-related processes. PGE2 is considered one of the primary factors driving tumor development and progression through stimulation of cell proliferation, survival, migration, and invasion [95,96]. Glioblastoma cell lines exhibit a high potential for TXB2 synthesis, with relative levels correlating with their migration rates and poor patient prognosis [97].

Additionally, COX-2-derived eicosanoids are known to modulate cytokine responses. PGE_2_, in particular, although pro-inflammatory, also has an immunomodulatory role: it elevates intracellular cAMP via EP2/EP4 receptors, which in macrophages is known to increase IL-10 production, while suppressing excessive TNF-α production [98]. Thus, the strong production of PGE_2_ in glioma cells exposed to NaF may contribute to the concomitant increase in IL-10 (reinforcing a feedback loop that suppresses inflammation). Our results indicate that TXB_2_ and PGE_2_ may be important downstream effectors of NaF action on glial cells—they are likely to be markers of oxidative/inflammatory enzyme activation and active participants in the regulation of the emerging cytokine network.

### 4.6. The Role of Fluorides in Regulating IFN-γ Levels

NaF at the concentrations used in this study influenced an increase in IFN-γ levels in both the culture medium and cells during long-term exposure. These results are intriguing, particularly when considered in the context of exposure duration. In this case, the reduction in IFN-γ levels during short-term exposure may be beneficial for the development and invasion of GBM cells. However, the immunomodulatory effects of IFN-γ on the GBM microenvironment remain unclear. Maintenance therapy using IFN-γ has not shown benefits for GBM patients [99], suggesting that our understanding of IFN-γ’s role in GBM, as well as its clinical significance, remains insufficient. The increase in IFN-γ observed during long-term NaF exposure might also have effects contrary to expectations, particularly since no cytotoxic effect of long-term NaF exposure was observed on U-87 cells at any of the tested concentrations [36]. Currently, there are no published data discussing the effects of fluorides on IFN-γ in GBM cells. However, isolated studies indicate that fluoride compounds can influence human immune cells in the context of IFN-γ. This has been demonstrated by measuring the effect of NaF on cytokine production, including IFN-γ, in human whole blood cells stimulated in vitro [100].

## 5. Conclusions

The results indicate that fluorides may exert multifaceted effects on glioma cells, leading to redox imbalance, activation of signaling pathways that favor tumor progression, and modulation of inflammatory processes (Table 1). These effects depend on both the NaF concentration and the duration of exposure. Increased ROS levels and changes in antioxidant enzyme activity may be key mechanisms of fluoride toxicity. Fluoride-induced oxidative stress may act as a trigger or enhancer of signaling pathways known to drive GBM progression, particularly its aggressive and invasive behavior.The involvement of inflammatory pathways such as NF-κB and the potential interaction with Wnt and AKT pathways are key areas for further investigation. Furthermore, the likely activation of the Wnt/β-catenin pathway and alterations in pro-inflammatory cytokine levels suggest a potential influence of NaF on glioma growth, invasiveness, and treatment resistance. The established oncogenic role of the Wnt/β-catenin pathway in GBM suggests that any activation of this pathway in response to fluoride-induced oxidative stress is most likely to contribute to tumor progression rather than act as a compensatory mechanism.

While these results do not allow for definitive conclusions, they highlight the need for broader studies on the mechanisms of fluoride toxicity in the context of central nervous system tumors.

## 6. Limitations and Challenges

While this study provides valuable in vitro data, a key limitation is that results obtained in cellular systems rarely fully reflect the complexity of the in vivo environment. In the human body, interactions are shaped by multiple cell types, the host immune system, and unique features of the tumor microenvironment. Additionally, the fluoride concentrations used in the experiment, although based on literature data, may differ across geographic regions and populations. Further studies, including animal models, are needed to confirm our observations.

## Figures and Tables

**Table 1:** Summary of sodium fluoride (NaF) effects on oxidative stress, Wnt signaling, and inflammatory mediators in glioblastoma U-87 cells.

Parameter	Marker	Effect	NaF (µM)	Exposure Time	Comments
Oxidative Stress	Reactive oxygen species (ROS)	↑↑	0.1–10	mainly LONG	ROS adaptation observed after 72 h
	Superoxide dismutase (SOD) activity	↓	0.1	SHORT	No significant effect at higher doses
	Catalase (CAT) activity	↓	5–10	SHORT and LONG	Observed trend without statistical significance
	Total glutathione (GSH/GSSG levels)	↔	all doses	SHORT and LONG	Stable glutathione system
Wnt Signaling Pathway	Intracellular β-catenin	↑	5–10	SHORT and LONG	Intracellular activation of the Wnt pathway
	Extracellular β-catenin (medium)	↔	all doses	SHORT and LONG	No significant extracellular release
Pro-inflammatory Markers	IFN-γ	↑	1–5	mainly LONG	Moderate extracellular secretion
	TNF-α	↑↑	1–10	SHORT and LONG	Higher levels intracellularly than extracellularly
	IL-1α	↑	mainly 10	mainly LONG	Extracellular secretion observed
	IL-1β	↑	1–10	SHORT and LONG	More evident extracellular secretion
	IL-6	↑↑	0.1–10	SHORT and LONG	Strongest response among IL cytokines
	TXB2	↑↑↑	mainly 1	SHORT and LONG	Prominent role of COX/eicosanoid pathway
	PGE2	↑↑↑	0.1–10	mainly LONG	Active secretion into the medium
Anti-inflammatory Markers	IL-4	↑	0.1–1	SHORT and LONG	Higher intracellular vs. medium levels
	IL-10	↑↑	0.1–5	LONG	Strong extracellular secretion despite decreased intracellular levels

Legend: ↑ moderate increase; ↑↑ significant increase; ↑↑↑ strong increase; ↓ moderate decrease; ↔ no significant change.

## Data Availability

The datasets generated during and analyzed during the current study are not publicly available due to they are part of PhD thesis but are available from the corresponding author on reasonable request.

## Abbreviations

2′:7′-DCFH-DA—2′ 7′-dichlorodihydrofluorescein diacetate; AKT—protein kinase B; AMP—adenosine-3′, 5′-monophosphate; Axl—AXL receptor tyrosine kinase; BBB—blood–brain barier; CAT—catalase; COX—cyclooxygenase; Dkk-1—Dickkopf; DMSO—dimethylsulfoxide; EGFR—epidermal growth factor receptor; ERK—extracellular sig-nal-regulated kinase; FBS—fetal bovine serum; GBM—glioblastoma; GST—glutathione transferase; GPx—glutathione peroxidase; GSH—glutathione; GSSG—oxidized form of glutathione; HIF-1—hypoxia-induced factor 1; HRP—horseradish peroxidase; IFN-γ—interferon γ; IGF1—insulin like growth factor 1; IL—interleukin; iNOS—inducible nitric oxide synthase; IQ—intelligence quotient; JAK—Janus kinase; JNK—c-Jun N-terminal kinases; MDA—malondialdehyde; MAPK—mitogen-activated protein; MCP-1—monocyte chemotactic protein-1; MTT—3-(4,5-dimethylthiazolyl-2)-2,5-diphenyl tetrazolium bromide; NF-κB—nuclear factor κB; NOX—NADPH oxidase; CNS—central nervous system; PBS—phosphate-buffered saline; PGE2—prostaglandin E2; PI3K—phosphatidylinositol 3-kinase; PKA—protein kinase type A; ROS—reactive oxygen species; SOD—superoxide dismutase; STAT3—signal transducers and activators of transcription; TAC—total antioxidant capacity; TGF-β—transforming growth factor β; TMZ –temozolomide; TNFα—tumor necrosis factor α; TXB2—thromboxane B2; Wnt—Wing-less-related integration site.
